# Initial observation or treatment for diabetic macular oedema with good visual acuity: two‐year outcomes comparison in routine clinical practice: data from the Fight Retinal Blindness! Registry

**DOI:** 10.1111/aos.14672

**Published:** 2020-11-16

**Authors:** Pierre‐Henry Gabrielle, Vuong Nguyen, Sanjeeb Bhandari, Hemal Mehta, Francesco Viola, Jennifer Arnold, Samantha Fraser‐Bell, Daniel Barthelmes, Catherine Creuzot‐Garcher, Mark Gillies

**Affiliations:** ^1^ Sydney Medical School Discipline of Ophthalmology Save Sight Institute The University of Sydney Sydney New South Wales Australia; ^2^ Department of Ophthalmology Dijon University Hospital Dijon France; ^3^ Department of Ophthalmology Royal Free London NHS Foundation Trust London UK; ^4^ IRCCS Cà Granda Foundation Maggiore Policlinico Hospital University of Milan Milan Italy; ^5^ Marsden Eye Research Sydney NSW Australia; ^6^ Department of Ophthalmology University Hospital Zurich University of Zurich Zurich Switzerland

**Keywords:** diabetic macular oedema, DME, good vision, good visual acuity, observation, visual acuity loss

## Abstract

**Purpose:**

To compare visual acuity (VA) change at 24 months in eyes with clinically significant DME (CSDME) and good VA initially treated *versus* initially observed in routine clinical practice.

**Methods:**

Retrospective analysis of treatment‐naïve eyes with CSDME and good VA (baseline VA ≥ 79 letters), with at least 24 months of follow‐up and initially managed with treatment (intravitreal treatment and/or macular laser) or observation with possible treatment after 4 months that were tracked in a prospectively designed observational registry.

**Results:**

We identified 150 eligible eyes (98 initially observed, 52 initially treated) of 130 patients. The proportion of eyes with at least a 5‐letter VA loss at 24 months was not significantly different between the groups: 65% with initial observation and 42% with initial treatment (p = 0.39). However, initially observed eyes were more likely to have a 10‐letter VA loss at 24 months (OR = 4.6, p = 0.022). Most of eyes in the initial observation group received at least one treatment (an intravitreal injection in 66% and macular laser in 20%) during the 24‐month period.

**Conclusions:**

The risk of 5 letters loss was similar between both management groups. However, initially observed eyes were more at risk of developing moderate visual loss and more than 80% of them required treatment over 24 months.

## Introduction

Several treatments, such as macular laser photocoagulation, surgery, intravitreal injections of VEGF inhibitors or steroids, have been proven to be effective for diabetic macular oedema (DME; Mitchell et al. 2011; Wells et al. 2016; Iglicki et al. 2019, 2018; Mello Filho et al. 2019; Zur et al. 2019). Recommendations from RCTs of treatments for clinically significant diabetic macular oedema (CSDME) do not apply to eyes with good vision as the trials generally only included eyes with impaired vision (Mitchell et al. 2011; Nguyen et al. 2012; Boyer et al. 2014; Heier et al. 2016; Wells et al. 2016). CSDME with good vision is, however, commonly encountered in clinical practice (Bressler et al. 2014).

Protocol V by DRCR.net was the first RCT to compare visual outcomes in centre‐involving DME (CI‐DME) with good visual acuity (≥79 letters read on a logarithm of the minimum angle of resolution [logMAR] VA chart, 20/25 Snellen equivalent) treated with prompt macular laser photocoagulation, prompt observation or prompt intravitreal aflibercept. It reported no significant difference in visual outcomes at two years among the three groups, suggesting that initially observed eyes may achieve similar outcomes to those of initially treated eyes with reduced risk of injection‐related adverse events and better cost‐effectiveness (Baker et al. 2019). However, outcomes of patients in routine clinical practice can differ from those in RCTs. Information on how best to treat eyes with DME and good vision is still limited (Busch et al. 2019). This study aimed to compare visual outcomes in these eyes 24 months after they had either received initial treatment versus initial observation with treatment possible after the first 4 months in routine clinical practice.

## Methods

### Design and setting

This was a retrospective analysis of data from a prospectively designed outcomes registry, the Fight Retinal Blindness! Project (Gillies et al. 2014). Treatment‐naïve eyes with clinically significant DME (CSDME; defined as DME meeting one of these criteria: oedema within 500 μm of the centre of the fovea or at least 1 disc area of swelling, any part of which is within disc diameter of the centre of fovea) with good VA (baseline VA ≥ 79 letters read on a logMAR chart or 20/25 Snellen equivalent) and at least 24 months of follow‐up were identified. Participants in this analysis included patients from practices in Australia, France, Italy, New Zealand, Switzerland and United Kingdom (UK). Institutional approval was obtained from the Royal Australian and New Zealand College of Ophthalmologists Human Research Ethics Committee, the French Institutional Review Board (IRB; Société Française d’Ophtalmologie IRB), the IRCCS Cà Granda Foundation Maggiore Policlinico Hospital Milan, the Cantonal Ethics Committee Zurich and the Caldicott Guardian at the Royal Free London NHS Foundation Trust. All patients gave their informed consent. Informed consent (“opt‐in consent”) was sought from patients in France, Italy, Switzerland and UK. Ethics committees in Australia and New Zealand approved the use of “opt‐out” patient consent. This study adhered to the tenets of the Declaration of Helsinki and followed the STROBE statements for reporting observational studies (von Elm et al. 2008).

### Data sources and measurements

The Fight Retinal Blindness! Registry has a module that collects data from eyes being managed for DME (Gillies et al. 2014). Patients tracked in the registry with treatment‐naïve CSDME and good VA could either be treated or observed based on the clinician's assessment and in consultation with the patient. One or both eyes were considered for the present analysis. Data were obtained prospectively from each clinical visit including the number of letters read on a logMAR VA ETDRS (the Early Treatment of Diabetic Retinopathy) Chart (best of uncorrected, corrected or pinhole), treatment given, the central subfield thickness (CST [µm]) measured using spectral‐domain optical coherence tomography (OCT), the presence of CSDME and whether or not it involved the centre of the fovea, procedures and ocular adverse events. The OCT scans were obtained using SD‐OCT devices: Heidelberg Spectralis (Heidelberg, Germany); Optovue Avanti (Fremont, California); Topcon 3D OCT‐2000 (Tokyo, Japan); and Cirrus Zeiss (Oberkochen, Germany). CST was calculated automatically using the same instrument in each centre at all visits. Demographic characteristics (age and gender), duration and types of diabetes, grading of diabetic retinopathy (DR) and previous treatments received (cataract surgery and vitrectomy) were recorded at baseline visit. Treatment decisions, including the choice of treatment, injection frequency and the number of macular laser treatments, were collected over the follow‐up period.

### Patient selection and groups

All eligible eyes with treatment‐naïve CSDME and good vision at baseline visit (as defined above) from 1 January 2010 to 31 March 2018 were considered for the study, thereby allowing at least 24 months of observation after the initial management decision. The 24‐month end‐point was considered to be the closest visit to 730 days of follow‐up ±30 days. Eligible eyes that received any intravitreal treatment, such as vascular endothelial growth factor (VEGF) inhibitors (ranibizumab [0.5mg Lucentis, Genentech Inc/Novartis], aflibercept [2mg Eylea, Bayer] or bevacizumab [1.25mg Avastin, Genentech Inc/Roche]) or steroid implant (dexamethasone [700µg Ozurdex intravitreal implant, Allergan) and/or macular laser photocoagulation at the baseline visit were defined as “initially treated eyes”. Initially observed eyes were defined as eligible eyes initially observed (i.e. no treatment received) for at least 4 months. There were no randomization for the management allocation and no specific management protocols, so the decision to treat or observe at baseline or during the follow‐up was determined by the physician based on symptoms, VA and OCT at the discretion of the physician in consultation with the patient, thereby reflecting routine clinical practice.

### Outcomes

The main outcome was the proportion of eyes with VA loss of at least 5 letters from baseline at 24 months. Secondary outcomes included the mean change in VA and CST from baseline at 24 months, the proportion of eyes with VA ≥ 84 letters (Snellen equivalent of 20/20), the proportion of eyes with VA loss of ≥10 and ≥15 letters from baseline at 24 months, time to receiving first treatment (any treatment, macular laser or intravitreal injection) in the observation group, median number of treatments, macular laser sessions and visits over 24 months in each group.

### Statistical analysis

Descriptive data were summarized using the mean (standard deviation), median (first and third quartiles) and percentages where appropriate. Eyes that completed at least 700 days of follow‐up were defined as “completers”. Demographic characteristics were compared between initial management groups using t‐tests, Wilcoxon rank‐sum tests, chi‐square tests or Fisher’s exact tests where appropriate. The proportion of eyes with different VA loss/gain and final VA of at least 84 letters at 24 months between groups was compared using logistic mixed‐effects regression. Regression analysis was adjusted for age, gender, VA/CST, lens status, CI‐DME and diabetic retinopathy severity at baseline as fixed effects, and nesting of outcomes within practitioners and patients with bilateral disease as random effects. We also compared VA and CST outcomes between groups over 24 months using mixed‐effects longitudinal generalized additive models with the interaction between initial management and time as the main predictor variable. Longitudinal models included all visits of included eyes and were adjusted for age, VA/CST, lens status, diabetic retinopathy severity and CI‐DME at baseline (fixed‐effects), and practice and intra‐patient correlation for bilateral cases (random‐effects). We used predictions from this model to plot VA/CST and the difference in the mean VA/CST change over 24 months in all eyes. Negative binomial or zero‐inflated Poisson regression models, adjusted for age, VA/CST, lens status, diabetic retinopathy and CI‐DME at baseline, practice and intra‐patient correlation with log days of follow‐up included as an offset variable was used as appropriate to compare the number of injections and visits between groups. Cox proportional hazards models adjusted for age, VA/CST, lens status, diabetic retinopathy severity and CI‐DME at baseline, practice and intra‐patient correlation were used to compare the time to the first treatment (any treatment, macular laser or intravitreal injection) over 24 months in the initial observation management group. Kaplan–Meier survival analysis was used to plot survival curves to first treatment, injection and laser over 24 months in the initial observation management group. A p‐Value of 0.05 or less was considered statistically significant. All analyses were conducted using R software version 3.6.3 (http://www.R‐project.org/) with the *glmmTMB* package (V0.2.3) for linear mixed‐effects and logistic mixed‐effects regression, the *emmeans* package (V1.3.3) for pairwise comparison of adjusted means, the *mgcv* package (V1.8–24) for the generalized additive (mixed) model computation, and the *coxme* package (V2.2–10) and the *survival* package (V 2.38) for the time to first treatment analysis.

## Results

### Study participants

**Fig. 1:**
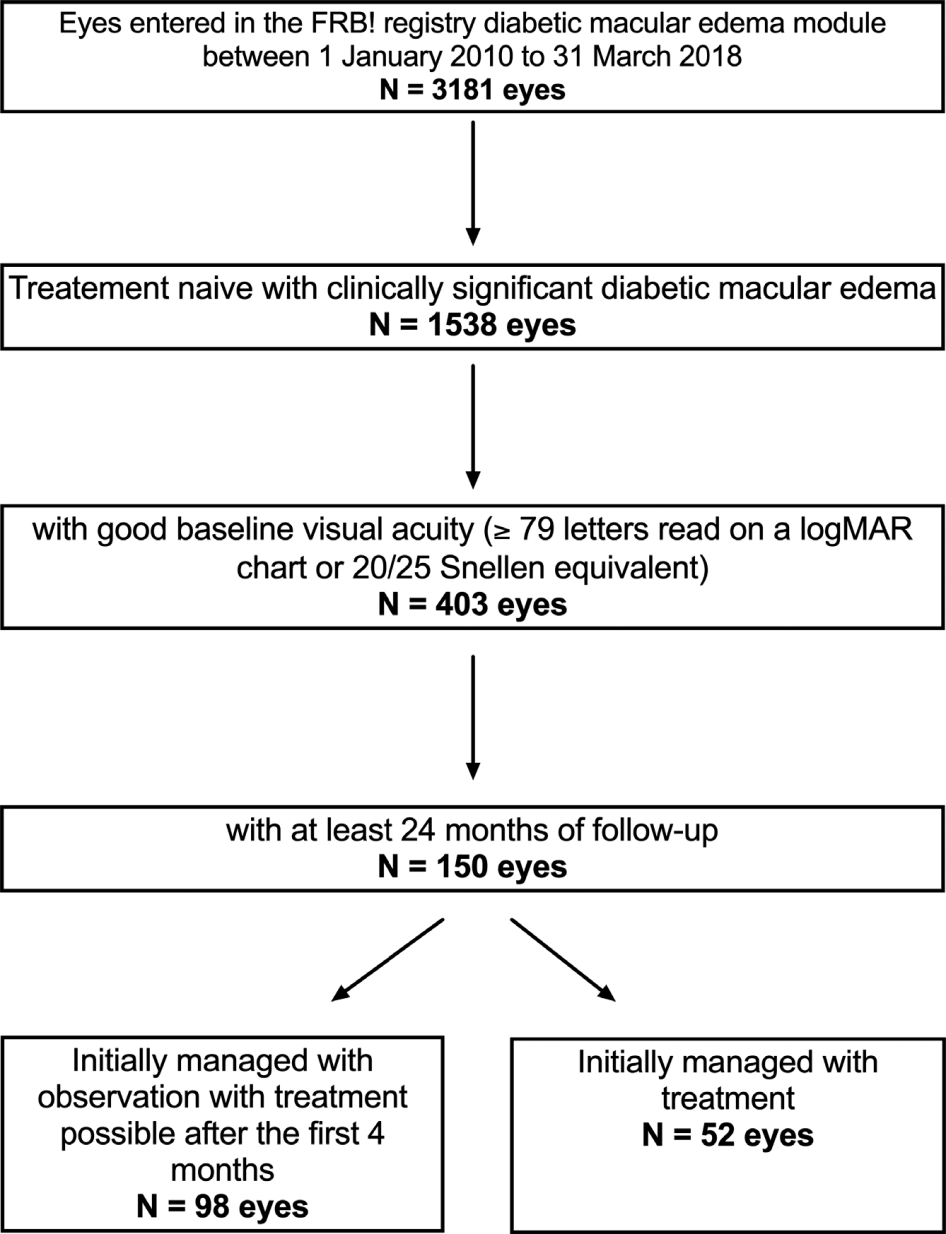
Flowchart showing the number of eyes remaining at each selection criterion.

A total of 150 treatment‐naïve CSDME eyes (98 initial observation and 52 initial treatment) of 130 patients from 1 January 2010 to 31 March 2018 were identified. The flowchart showing the number of eyes at each selection criterion is shown in Fig. 1. Table 1 summarizes the baseline characteristics of the eyes in each of the groups. Overall, the mean (SD) age was 60 (11) and 32% were women. Eyes initially managed with observation had better baseline VA (mean 84 versus 82 letters; p < 0.01), thinner CST (mean 308 versus 338 µm; p = 0.012) and less likely to have CSDME which involved the foveal centre (63% versus 89%; p < 0.01) than initially treated eyes. The initial observation group was significantly older (62 versus 57 years in the treatment group; p = 0.010) and had less type 1 diabetes (4% versus 20% in the treatment group; p < 0.01).

**Table 1:** Baseline characteristics of the study groups.

	Overall	Initial observation	Initial treatment	p‐Value
Eyes, *n*	150	98	52	
Patients, *n*	130	83	50	
Female, *n* (%)	42 (32)	31 (37)	13 (26)	0.16
Age years, mean (SD)	60 (11)	62 (9)	57 (13)	**0.010**
Type 1 Diabetes, *n* (%)	13 (10)	3 (4)	10 (20)	**<0.01**
Diabetes duration years, mean (SD)	14 (10)	14 (9)	15 (11)	0.72
Lens status (phakic), *n* (%)	136 (91)	90 (92)	46 (89)	0.70
Diabetic Retinopathy grades, %				
Mild NPDR	17	17	17	0.21
Moderate NPDR	43	39	50	
Severe NPDR	33	39	23	
PDR – Low Risk	5	4	6	
PDR – High Risk	2	1	4	
Visual acuity logMAR letters, mean (SD)	83 (3)	84 (3)	82 (3)	**<0.01**
Central subfield thickness μm, mean (SD)	320 (67)	308 (62)	338 (70)	**0.012**
Type of DME, %				
Centre‐involving CSDME	72	63	89	**<0.01**
Non‐centre‐involving CSDME	28	37	11	
Initial management, *n* (%)				
Observation	98 (65)	98 (100)	–	–
Bevacizumab	8 (5)	–	8 (15)	
Ranibizumab	22 (14)	–	22 (42)	
Aflibercept	9 (6)	–	9 (18)	
Dexamethasone implant	1 (1)	–	1 (2)	
Macular laser photocoagulation	12 (9)	–	12 (23)	

CSDME = Clinically Significant Diabetic Macular Oedema; CST = Central Subfield Thickness; DME = Diabetic Macular Oedema; n = Number; NPDR = Non‐Proliferative Diabetic Retinopathy; PDR = Proliferative Diabetic Retinopathy; SD = Standard Deviation; VA = Visual Acuity (logMAR letters).

Significant p‐values are highlighted in bold.

### Visual outcomes at 24 months

**Fig. 2:**
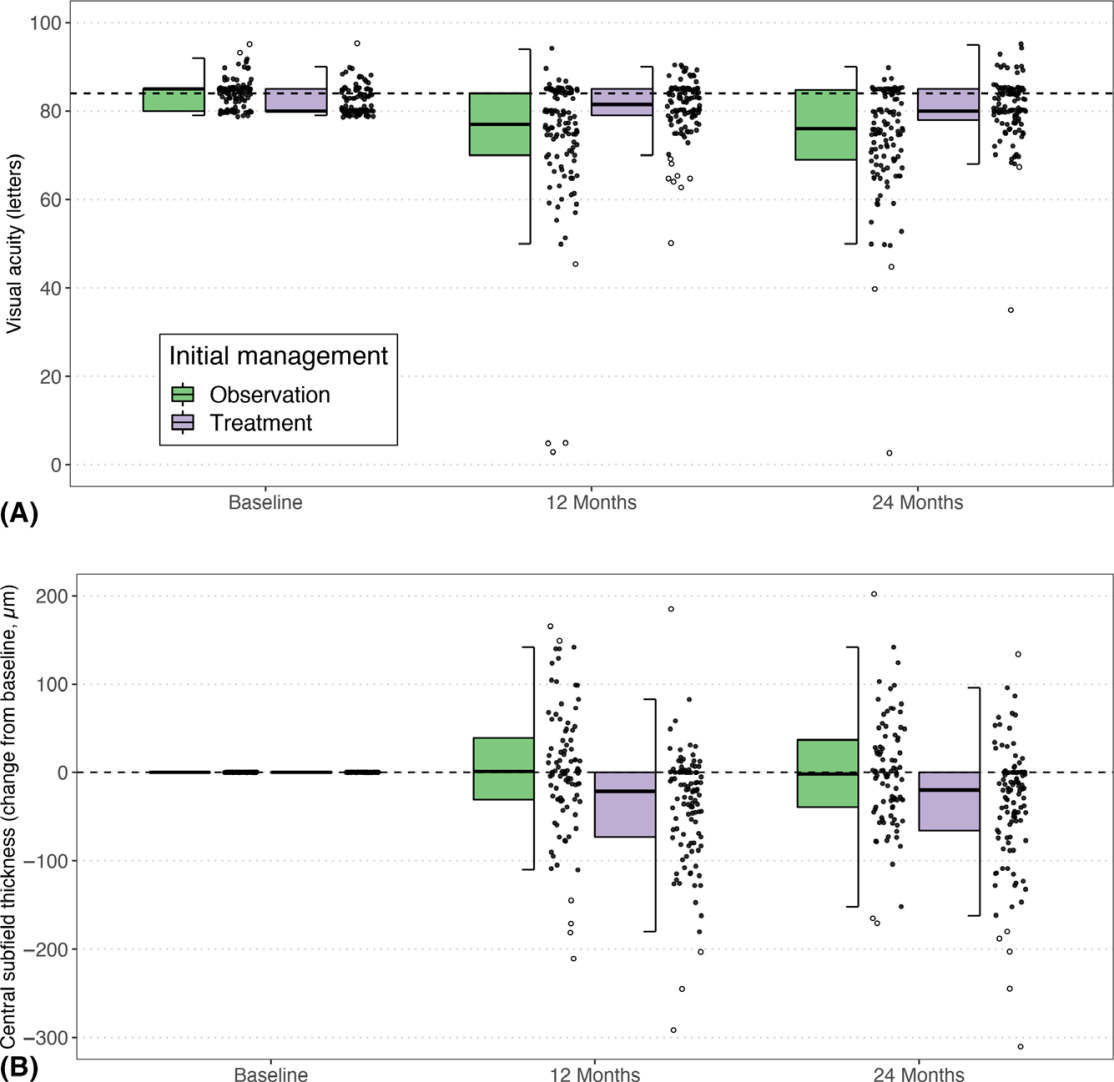
(A) Visual acuity and (B) change in central subfield thickness at 24 months. For each box‐and‐whisker plot with jitter, the horizontal bar within the box represents median; top and bottom of box, interquartile range; Upper and lower whisker extends to the closest observed data point below the upper or above the lower quartile plus 1.5 times the interquartile range. Jitters and Outlying values are plotted as black spots and circles, respectively. Values in panel A at or above the horizontal dashed line (84 letters) represent visual acuity of 20/20 or better.

**Fig. 3:**
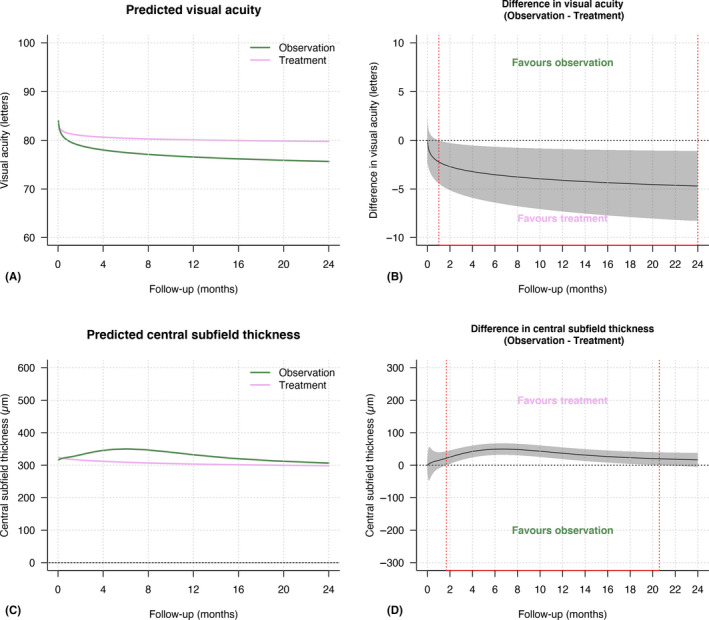
Line graphs showing (A) the mean predicted visual acuity in logMAR letters; (B) the difference in the mean change in VA between initial observation (pink) and initial treatment (green); (C) the mean predicted central subfield thickness (CST, solid lines) and (D) the difference in mean change in CST between initial observed and treated eyes over 24 months. The grey‐shaded area in figures B and D represents the 95% confidence interval. Red‐dashed lines in B and D indicate areas where the 95% confidence interval does not intersect with 0.

The percentage of eyes with at least a 5‐letter VA loss at 24 months (primary outcome) between initial observation and initial treatment was 65% versus 42% (Odds Ratio [OR] = 1.6 [0.5, 5.1], p < 0.39; Table 2). Eyes in the initial observation group were more likely to have a 10‐letter and 15‐letter VA loss at 24 months (OR = 4.6 [1.3, 17.0], p = 0.022 and OR = 18.5 [0.8,410.0], p = 0.065, respectively). Figure 2A describes the crude mean (SD) VA at 12 and 24 months. The proportion of eyes with VA ≥ 84 letters (Snellen equivalent of 20/20) at 24 months was 29% with initial observation and 35% with initial treatment (OR = 0.8 [0.3, 2.6], p = 0.77). Eyes that were initially treated seemed more likely to have driving level vision (VA ≥ 69 letters logMAR or 20/40 Snellen equivalent) after 24 months of treatment (92% versus 72% for initially observed eyes, p = 0.09; Table 2). The adjusted mean VA over 24 months, using longitudinal generalized additive models, is shown in Fig. 3A. The adjusted mean (95% CI) difference in the VA change was significantly in favour of initial treatment for most of the 24 months (−8 [−10, −5] for observation versus −3 [−6, 0] letters for treatment, p = 0.020; Fig. 3B and Table 2).

**Table 2:** 24 month outcomes.

Outcomes	Observed data		Observation versus treatment adjusted group comparison	
	Initial observation	Initial treatment	Odds ratio, mean difference or ratio (95% CI)	p‐Value
*Primary outcomes*				
≥5‐letter VA loss, *n* (%)	64 (65)	22 (42)	1.6 (0.5, 5.1) ^†^	0.39
*Secondary outcomes*				
Baseline VA letters, mean (SD)	84 (3)	82 (3)		
Baseline VA Snellen equivalent, mean	20/25	20/25		
VA change from baseline to 24 months letters, crude mean (95% CI)	−10 (−13, −8)	−3 (−6, 0)		**<0.01**
VA change from baseline to 24 months letters, adjusted mean (95% CI)	−8 (−10, −5)	−3 (−6, 0)	−4 (−8, −1)^‡^	**0.020**
≥10‐letter loss from baseline, *n* (%)	39 (40)	10 (19)	4.6 (1.3, 17.0)^†^	**0.022**
≥15‐letter loss from baseline, *n* (%)	29 (30)	2 (4)	18.5 (0.8, 410.0)^†^	0.065
VA at 24 months letters, mean (SD)	74 (13)	79 (9)		
VA at 24 months Snellen equivalent, mean	20/32	20/25		
≥84 letters (20/20 or better), *n* (%)	28 (29)	18 (35)	0.8 (0.3, 2.6)^†^	0.77
≥69 letters (20/40 or better), *n* (%)	73 (75)	48 (92)	0.3 (0.1, 1.2)^†^	0.09
Baseline CST μm, mean (SD)	307.9 (61.8)	338.4 (69.8)		
CST change from baseline to 24 months μm, crude mean (95% CI)	0 (−19, 19)	−32 (−56, −9)		**0.033**
CST change from baseline to 24 months μm, adjusted mean (95% CI)	−16 (−34, 2)	−24 (−43, −6)	+8 (−14, 30)^‡^	**<0.01**
Injections, median (Q1, Q3)	6 (3, 10)	6 (3, 10)	1.1 (0.8, 1.4)^§^	0.77
Proportion of eyes receiving any treatment in the observation group	77 (79)	NA		
Proportion of eyes receiving at least one injection in the observation group, *n* (%)	65 (66)	NA		
Time until first injection days, median (Q1, Q3)	554 (322, 827)	–		
VA at first injection letters, mean (SD)	72 (11)	–		
VA change at first injection letters, mean (SD)	−12 (11)	–		
<5‐letters loss, *n* (%)	21 (21)	–		
≥5 and <10‐letters loss, *n* (%)	13 (13)	–		
≥10 letters loss, *n* (%)	31 (32)	–		
Proportion of eyes receiving at least one laser photocoagulation in the observation group, *n* (%)	20 (20)	NA		
Time until first laser days, median (Q1, Q3)	320 (225, 607)	–		
VA at first laser letters, mean (SD)	−8 (9)	–		
Laser photocoagulation sessions, median (Q1, Q3)	0 (0, 0)	0 (0, 1)	0.8 (0.4, 1.5)^§^	0.48
Visits, median (Q1, Q3)	17 (12, 21)	13 (9, 18)	1.6 (1.3, 2.0)^§^	**<0.01**

CI = Confidence Interval; CST = Central subfield thickness; *n* = Number; Q1 = First Quantile; Q3 = Third Quantile; SD = Standard Deviation; VA = Visual Acuity.

Significant p‐values are highlighted in bold.

^†^ Calculated from logistic mixed‐effects regression models adjusting for age, VA/CST, lens status, centre‐involving diabetic macular oedema, grade of diabetic retinopathy at baseline (fixed effects), and practice and intra‐patient correlation for bilateral cases (random effects).

^‡^ Calculated from non‐linear mixed‐effects regression models adjusting for age, VA/CST, lens status, centre‐involving diabetic macular oedema, grade of diabetic retinopathy at baseline (fixed effects), and practice and intra‐patient correlation for bilateral cases (random effects).

^§^ Adjusted ratio (95% CI) of number of injections, laser photocoagulations or visits between observation versus treatment group. It was calculated from Poisson regression models adjusting for age, VA/CST, lens status, centre‐involving diabetic macular oedema, grade of diabetic retinopathy at baseline (fixed effects), and practice and intra‐patient correlation for bilateral cases (random effects).

### Macular thickness at 24 months

Figure 2B reports the crude mean (SD) CST change at 12 and 24 months. The adjusted mean CST change over 24 months using adjusted longitudinal generalized additive models is shown in Fig. 3C. The adjusted mean (95% CI) difference in the CST change was significantly in favour of initial treatment from 2 to 20 months of follow‐up but was similar by the end of the 24‐month follow‐up (−16 [−34, 2] for observation versus −24 [−43, −6] µm for treatment, p < 0.01; Fig. 3D and Table 2).

### Treatments and visits over 24 months

**Fig. 4:**
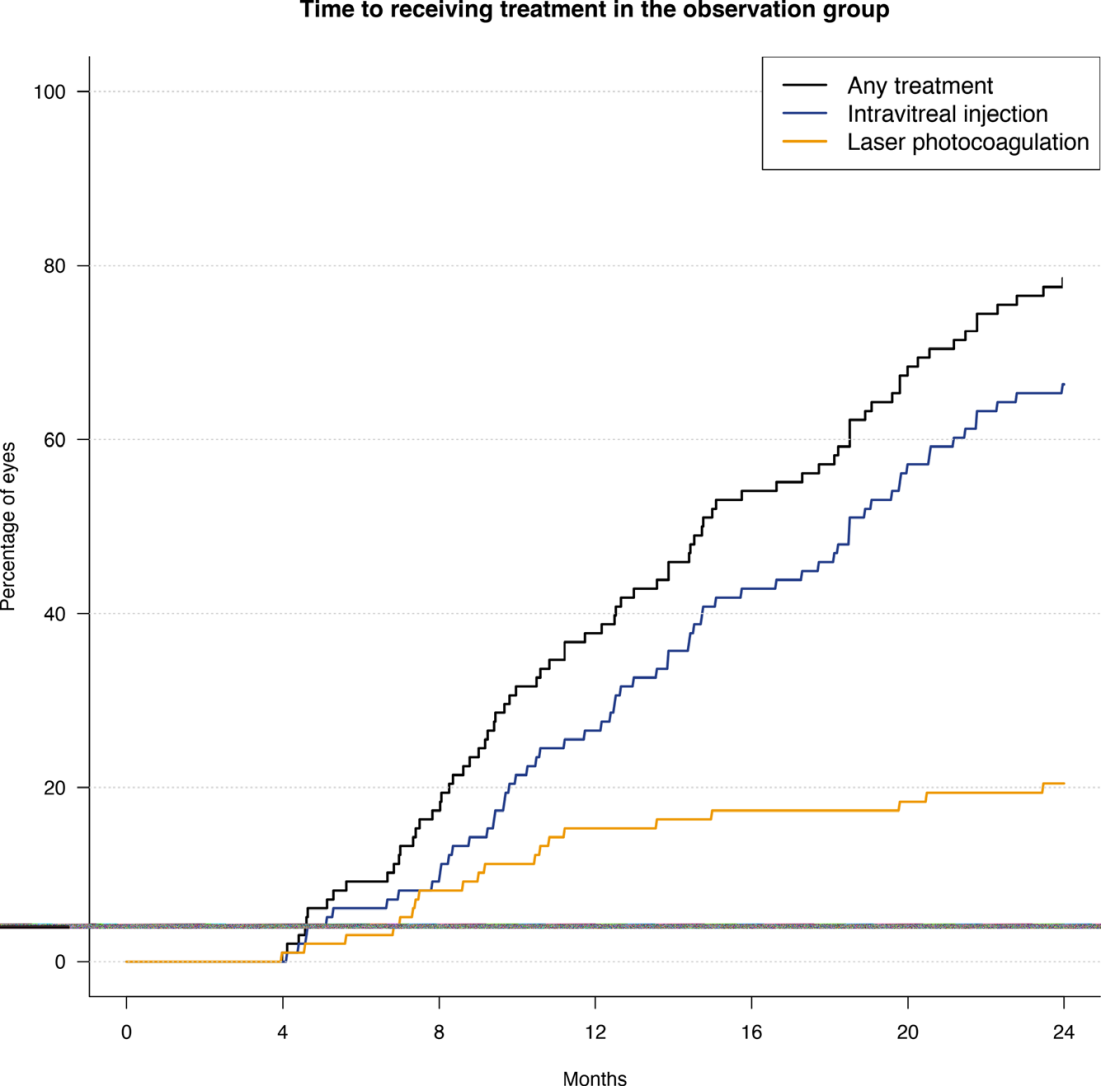
Kaplan–Meier plots for time to first treatment, intravitreal injection and laser photocoagulation in the initial observation group.

At least one intravitreal injection was given to 66% of eyes in the initial observation group over 24 months, 20% received macular laser and 13% received both (Fig. 4). Eyes in the observation group that received at least one intravitreal injection had a mean (SD) change in VA of −12 (11) letters from baseline to the time of the injection at a median (Q1, Q3) time of 554 (322, 827) days (Table 2). Eyes with thicker maculae at baseline were more likely to receive an intravitreal injection over 2 years (hazard ratio [HR] = 2.3 [1.1, 4.9] for every 100 μm increase of CST, p = 0.027).

The median (Q1, Q3) number of intravitreal injections and macular laser sessions over 24 months was similar between groups (6 (3, 10) versus 6 (3, 10) injections, p = 0.97 and 0 (0, 0) versus 0 (0, 1) laser sessions, p = 0.20 in the initial observation and initial treatment groups, respectively). The vast majority (97%) of intravitreal treatment in both groups were with VEGF inhibitors while the dexamethasone implant accounted for the remaining 3%. However, initially observed eyes had a significantly higher median number of visits over 24 months (17 (12, 21) versus 13 (9, 18) visits for initially treated eyes, p < 0.01; Table 2).

## Discussion

We used the FRB! international observational outcomes database to explore the effect of initial management on visual outcomes in treatment‐naïve eyes with CSDME (including centre‐involving and non‐centre‐involving DME) and good vision (baseline VA ≥ 79 letters or 20/25 Snellen equivalent) in routine clinical practice. The proportion of eyes developing 5‐letter VA loss at 24 months was not significantly different between eyes initially treated and eyes initially observed for at least 4 months. This finding is consistent with the DRCRnet protocol V that found no significant difference in the rates of 5 letter VA loss over 2 years between the following 3 groups: initial observation plus aflibercept only if VA decreased, initial focal/grid laser plus aflibercept only if VA decreased or prompt aflibercept (Baker et al. 2019).

The secondary outcomes in our study may be of more clinical interest. We found that eyes initially observed had significantly greater loss of adjusted mean change in VA, including significantly higher risk of 10‐letter VA loss over 24 months. This differs from the results of DRCR.net Protocol V, which did not find any significant difference regarding the mean change in VA and the proportion of 10‐letter vision loss at 2 years. The study design, different inclusion/exclusion criteria and a less rigorous protocol of treatment and follow‐up in a routine clinical practice setting may be reasons for the poorer visual outcomes that we found in the initial observation group (Holekamp et al. 2018). Visual outcomes in observational studies have generally been inferior to those reported by RCTs (Patrao et al. 2016; Egan et al. 2017; Bhandari et al. 2019; Lukic et al. 2019).

At least one intravitreal injection was given to 66% of eyes in the initial observation group over 24 months, 20% received laser photocoagulation and 13% received both. This is higher than reported by the Protocol V study, with only 40% of the observation and 30% of the laser photocoagulation groups requiring at least one injection of aflibercept over 2 years (Baker et al. 2019). We included only eyes with DME that were treatment‐naïve, whereas protocol V also included pretreated eyes (approximately 15% of the observation and laser photocoagulation cohorts; Baker et al. 2019). Those eyes may have benefited from the effect of previous laser or intravitreal treatment and might be less likely to require further treatment. A recent secondary analysis of Protocol V showed that initially observed DME eyes with contralateral eye concomitantly treated with intravitreal VEGF inhibitors for DME are at increased risk of receiving an early injection (Glassman et al. 2020). Three patients were in that case in our study, so it is unlikely that those patients substantially increased the likelihood of receiving an injection.

The mean (SD) loss of VA until the first injection in the observation group was 12 (12) letters. Intravitreal treatment was started with relatively high VA loss in our routine clinical practice observational study but similar to the Protocol V study treatment initiation criteria (i.e., at least 10 letters VA loss from baseline at one visit). A retrospective study comparing one year outcomes of treated and untreated DME eyes with good VA reported that continuing observation if VA decreases ≥5 letters within 6 months led to worse visual outcome at one year compared with initiating treatment (Busch et al. 2019). The introduction of treatment was based on symptoms, VA and OCT at the discretion of the physician in consultation with the patient agreement, thereby representing routine clinical practice. Several studies have suggested that compliance and adherence to treatment may be difficult in patients with DME in routine clinical practice due to a range of factors including visit and treatment burden, not just of the eye disease but of diabetes in general and its comorbidities (Schnabel et al. 2016), which may result in worse long‐term visual outcomes (Ehlken et al. 2018; Weiss et al. 2018).

It is worth noting that the initial treatment group did not experience a clinically significant visual gain and did not have a significantly higher proportion of eyes with VA ≥ 84 letters over 24 months than the initial observation group. This is probably due to a ceiling effect that is usually observed in eyes with DME and good vision (Mitchell et al. 2011; Nguyen et al. 2012; Korobelnik et al. 2014; Heier et al. 2016; Wells et al. 2016; Busch et al. 2019). However, more initially treated eyes had driving vision after 24 months of treatment. Early treatment may therefore reduce the risk of loss of independence, which may have a dramatic impact on the quality of life.

The median number of injections in both groups over 24 months was remarkably similar even though treatment was delayed for at least 4 months in the observation group. A similar observation was made by the DRCR.net protocol V study group (Baker et al. 2019). Eyes in the initial observation group had significantly more visits over 24 months. These findings suggest that a delay in the initiation of treatment results in a requirement of more intensive treatment and monitoring to maintain good vision. The inconvenience of starting treatment early may thus be offset by reducing the long‐term visit burden for both patients and physicians. The proposed benefits of deferring treatment, such as reduced risk of endophthalmitis and the economic benefit for the health care system in savings on drug and procedures, deserve to be reconsidered (Baker et al. 2019). Further studies are warranted to address these concerns.

We reported that initially treated eyes led to better anatomical outcomes compared to observation with a greater improvement of CST during most of 24 months. We also found that initially observed eyes with thicker CST at baseline had an increased risk of having at least one intravitreal injection over 24 months as recently described in a secondary analysis from DRCR protocol V (Glassman et al. 2020).

This study has several strengths. Observational studies provide data on the ability of an intervention to achieve its intended purpose in clinical practice. Our data are representative of a wide variety of international practices and practitioners. Though there is variability in the quality of data in observational studies, the FRB! system includes quality assurance measures that preclude out of range and missing data (Gillies et al. 2014). Well‐designed observational studies are unlikely to overestimate the effect of treatment compared with the results of randomized clinical trials (Concato et al. 2000).

We acknowledge several limitations that are mostly inherent in observational studies. First, treatment decisions in routine clinical practice are made without reference from a guided management protocol or reading centre and are likely to differ among physicians and centres, particularly in different countries. We included nesting of outcomes within practitioners in our models to help account for these effects. Second, a lack of prospective randomization resulted in significant differences in baseline characteristics between the initial observation and initial treatment groups. However, we have attempted to control for these imbalances by adjusting the statistical analysis for potential unbalanced confounders such as age, gender, lens status, VA/CST, CI‐DME and the severity of DR at baseline although we did not have data on glycaemic and blood pressure control which may have influenced our results. Third, our study may have been underpowered due to the relatively small sample sizes, though we were able to detect statistically and clinically significant differences in a number of secondary outcomes. A priori power analysis calculation for detecting a 25% difference in the proportion of 5‐letter VA loss over 24 months with 80% power (α error = 0.05) via logistic regression suggested a sample size of 149 (ratio of 2:1 between groups) would be sufficient. Fourth, we included all types of CSDME in our studies, whereas Protocol V included only CI‐DME. We adjusted outcomes to control for baseline foveal involvement. A sensitivity analysis on the cohort of eyes with CI‐DME did not affect the main findings of the primary analysis (Tables S1 and S2, Figs [Link], [Link], [Link]). Overall mean CST was similar to Protocol V study (320 versus 311 µm in Protocol V; Baker et al. 2019). Fifth, we were unable to compare the risk of diabetic retinopathy progression between both strategies (Gross et al. 2015; Iglicki et al. 2018) and to address the influence of possible functional predictive imaging factors on the results (Busch et al. 2020; Zur et al. 2020).

In conclusion, CSDME eyes with good vision initially managed with treatment versus observation with possible treatment after 4 months had similar rates of 5‐letter visual loss over 24 months. However, initially observed eyes were more at risk of developing moderate visual loss and more than 80% of them subsequently required treatment with more follow‐up visits over 24 months. Initiating treatment may be a better management option for good vision DME in the case of patients with weak adherence and compliance since it decreases the risk of visual loss and reduces the patient’s management burden from diabetes and associated comorbidities. The development of less invasive or more durable treatments may further tilt the balance in favour of initiating treatment earlier.

## Supporting information


**Fig. S1.** (A) Visual acuity and (B) change in central subfield thickness at 24 months when only eyes with center‐involving diabetic macular edema are included.

**Fig. S2.** Line graphs showing (A) the mean predicted visual acuity in logMAR letters; (B) the difference in the mean change in VA between initial observation (pink) and initial treatment (green); (C) the mean predicted central subfield thickness (CST, solid lines) and (D) the difference in mean change in CST between initial observed and treated eyes over 24 months when only eyes with center‐involving diabetic macular edema are included.

**Fig. S3.** Kaplan–Meier plots for time to first treatment, intravitreal injection and laser photocoagulation in the initial observation group when only eyes with center‐involving diabetic macular edema are included.

**Table S1.** Baseline characteristics of the study groups when only eyes with center‐involving diabetic macular edema are included.

**Table S2.** 24‐month outcomes when only eyes with center‐involving diabetic macular edema are included.

## References

[aos14672-bib-0001] Baker CW , Glassman AR , Beaulieu WT et al. (2019): Effect of initial management with aflibercept vs laser photocoagulation vs observation on vision loss among patients with diabetic macular Edema involving the center of the macula and good visual acuity: a randomized clinical trial. JAMA 321: 1880–1894. 31037289 10.1001/jama.2019.5790 PMC6537845

[aos14672-bib-0002] Bhandari S , Nguyen V , Fraser‐Bell S et al. (2019): Ranibizumab or aflibercept for diabetic macular edema: comparison of 1‐year outcomes from the fight retinal blindness! Registry. Ophthalmology 127: 608–615. 31932092 10.1016/j.ophtha.2019.11.018

[aos14672-bib-0003] Boyer DS , Yoon YH , Belfort R et al. (2014): Three‐year, randomized, sham‐controlled trial of dexamethasone intravitreal implant in patients with diabetic macular edema. Ophthalmology 121: 1904–1914. 24907062 10.1016/j.ophtha.2014.04.024

[aos14672-bib-0004] Bressler NM , Varma R , Doan QV et al. (2014): Underuse of the health care system by persons with diabetes mellitus and diabetic macular edema in the United States. JAMA Ophthalmol 132: 168–173. 24357541 10.1001/jamaophthalmol.2013.6426 PMC4576971

[aos14672-bib-0005] Busch C , Fraser‐Bell S , Zur D et al. (2019): Real‐world outcomes of observation and treatment in diabetic macular edema with very good visual acuity: the OBTAIN study. Acta Diabetol 56: 777–784. 30903434 10.1007/s00592-019-01310-z PMC6558052

[aos14672-bib-0006] Busch C , Okada M , Zur D et al. (2020): Baseline predictors for visual acuity loss during observation in diabetic macular oedema with good baseline visual acuity. Acta Ophthalmol Epub ahead of print. 10.1111/aos.14390 32115886

[aos14672-bib-0007] Concato J , Shah N & Horwitz RI (2000): Randomized, controlled trials, observational studies, and the hierarchy of research designs. N Engl J Med 342: 1887–1892. 10861325 10.1056/NEJM200006223422507 PMC1557642

[aos14672-bib-0008] Egan C , Zhu H , Lee A et al. (2017): The United Kingdom Diabetic Retinopathy Electronic Medical Record Users Group, Report 1: baseline characteristics and visual acuity outcomes in eyes treated with intravitreal injections of ranibizumab for diabetic macular oedema. Br J Ophthalmol 101: 75–80. 27965262 10.1136/bjophthalmol-2016-309313

[aos14672-bib-0009] Ehlken C , Helms M , Böhringer D , Agostini HT & Stahl A (2018): Association of treatment adherence with real‐life VA outcomes in AMD, DME, and BRVO patients. Clin Ophthalmol 12: 13–20. 29339917 10.2147/OPTH.S151611 PMC5745150

[aos14672-bib-0010] von Elm E , Altman DG , Egger M , Pocock SJ , Gøtzsche PC , Vandenbroucke JP & STROBE Initiative (2008): The Strengthening the Reporting of Observational Studies in Epidemiology (STROBE) statement: guidelines for reporting observational studies. J Clin Epidemiol 61: 344–349. 18313558 10.1016/j.jclinepi.2007.11.008

[aos14672-bib-0011] Gillies MC , Walton R , Liong J et al. (2014): Efficient capture of high‐quality data on outcomes of treatment for macular diseases: the fight retinal blindness! Project. Retina 34: 188–195. 23836194 10.1097/IAE.0b013e318296b271

[aos14672-bib-0012] Glassman AR , Baker CW , Beaulieu WT et al. (2020): Assessment of the DRCR retina network approach to management with initial observation for eyes with center‐involved diabetic macular edema and good visual acuity: a secondary analysis of a randomized clinical trial. JAMA Ophthalmol 138: 341–349. 32077907 10.1001/jamaophthalmol.2019.6035 PMC7042938

[aos14672-bib-0013] Gross JG , Glassman AR , Jampol LM et al. (2015): Panretinal photocoagulation vs intravitreous ranibizumab for proliferative diabetic retinopathy: a randomized clinical trial. JAMA 314: 2137–2146. 26565927 10.1001/jama.2015.15217 PMC5567801

[aos14672-bib-0014] Heier JS , Korobelnik J‐F , Brown DM et al. (2016): Intravitreal aflibercept for diabetic macular edema: 148‐Week results from the VISTA and VIVID studies. Ophthalmology 123: 2376–2385. 27651226 10.1016/j.ophtha.2016.07.032

[aos14672-bib-0015] Holekamp NM , Campbell J , Almony A , Ingraham H , Marks S , Chandwani H , Cole AL & Kiss S (2018): Vision outcomes following anti‐vascular endothelial growth factor treatment of diabetic macular edema in clinical practice. Am J Ophthalmol 191: 83–91. 29684329 10.1016/j.ajo.2018.04.010

[aos14672-bib-0016] Iglicki M , Busch C , Zur D et al. (2019): Dexamethasone implant for diabetic macular edema in naive compared with refractory eyes: the International Retina Group real‐life 24‐month multicenter study. The IRGREL‐DEX Study. Retina 39: 44–51. 29697589 10.1097/IAE.0000000000002196

[aos14672-bib-0017] Iglicki M , Lavaque A , Ozimek M et al. (2018): Biomarkers and predictors for functional and anatomic outcomes for small gauge pars plana vitrectomy and peeling of the internal limiting membrane in naïve diabetic macular edema: The VITAL Study. PLoS One 13: e0200365. 29995929 10.1371/journal.pone.0200365 PMC6040739

[aos14672-bib-0018] Iglicki M , Zur D , Busch C , Okada M & Loewenstein A (2018): Progression of diabetic retinopathy severity after treatment with dexamethasone implant: a 24‐month cohort study the ‘DR‐Pro‐DEX Study’. Acta Diabetol 55: 541–547. 29497837 10.1007/s00592-018-1117-z

[aos14672-bib-0019] Korobelnik J‐F , Do DV , Schmidt‐Erfurth U et al. (2014): Intravitreal aflibercept for diabetic macular edema. Ophthalmology 121: 2247–2254. 25012934 10.1016/j.ophtha.2014.05.006

[aos14672-bib-0020] Lukic M , Williams G , Shalchi Z et al. (2019): Intravitreal aflibercept for diabetic macular oedema: Moorfields’ real‐world 12‐month visual acuity and anatomical outcomes. Eur J Ophthalmol 30: 557–562. 30808179 10.1177/1120672119833270

[aos14672-bib-0021] Mello Filho P , Andrade G , Maia A et al. (2019): Effectiveness and safety of intravitreal dexamethasone implant (Ozurdex) in patients with diabetic macular edema: a real‐world experience. Ophthalmologica 241: 9–16. 30408801 10.1159/000492132

[aos14672-bib-0022] Mitchell P , Bandello F , Schmidt‐Erfurth U et al. (2011): The RESTORE study: ranibizumab monotherapy or combined with laser versus laser monotherapy for diabetic macular edema. Ophthalmology 118: 615–625. 21459215 10.1016/j.ophtha.2011.01.031

[aos14672-bib-0023] Nguyen QD , Brown DM , Marcus DM et al. (2012): Ranibizumab for diabetic macular edema: results from 2 phase III randomized trials: RISE and RIDE. Ophthalmology 119: 789–801. 22330964 10.1016/j.ophtha.2011.12.039

[aos14672-bib-0024] Patrao NV , Antao S , Egan C et al. (2016): Real‐world outcomes of ranibizumab treatment for diabetic macular edema in a united kingdom national health service setting. Am J Ophthalmol 172: 51–57. 27637784 10.1016/j.ajo.2016.09.002

[aos14672-bib-0025] Schnabel R , Tan J , Cullip T , Guinan G & Cameron C (2016): Barriers to intravitreal injection therapy adherence in DME patients in Australia. Value Health 19: A844.

[aos14672-bib-0026] Weiss M , Sim DA , Herold T et al. (2018): Compliance and adherence of patients with diabetic macular edema to intravitreal anti‐vascular endothelial growth factor therapy in daily practice. Retina 38: 2293–2300. 29068914 10.1097/IAE.0000000000001892

[aos14672-bib-0027] Wells JA , Glassman AR , Ayala AR et al. (2016): Aflibercept, bevacizumab, or ranibizumab for diabetic macular edema: two‐year results from a comparative effectiveness randomized clinical trial. Ophthalmology 123: 1351–1359. 26935357 10.1016/j.ophtha.2016.02.022 PMC4877252

[aos14672-bib-0028] Wells JA , Glassman AR , Jampol LM et al. (2016): Association of baseline visual acuity and retinal thickness with 1‐year efficacy of aflibercept, bevacizumab, and ranibizumab for diabetic macular edema. JAMA Ophthalmol 134: 127–134. 26605836 10.1001/jamaophthalmol.2015.4599 PMC5567793

[aos14672-bib-0029] Zur D , Iglicki M & Loewenstein A (2019): The role of steroids in the management of diabetic macular edema. Ophthalmic Res 62: 231–236. 31048580 10.1159/000499540

[aos14672-bib-0030] Zur D , Iglicki M , Sala‐Puigdollers A et al. (2020): Disorganization of retinal inner layers as a biomarker in patients with diabetic macular oedema treated with dexamethasone implant. Acta Ophthalmol 98: e217–e223. 31421028 10.1111/aos.14230

