# Supplementary material for: Initial observation or treatment for diabetic macular oedema with good visual acuity: two‐year outcomes comparison in routine clinical practice: data from the Fight Retinal Blindness! Registry
Source: Acta Ophthalmol. 2020 Nov 16;100(3):285–94. doi: 10.1111/aos.14672 (PMC9290829; doi:10.1111/aos.14672)
Supplement: Supplementary file 1 — Fig. S1. (A) Visual acuity and (B) change in central subfield thickness at 24 months when only eyes with center‐involving diabetic macular edema are included. [file AOS-100-285-s002.pdf]

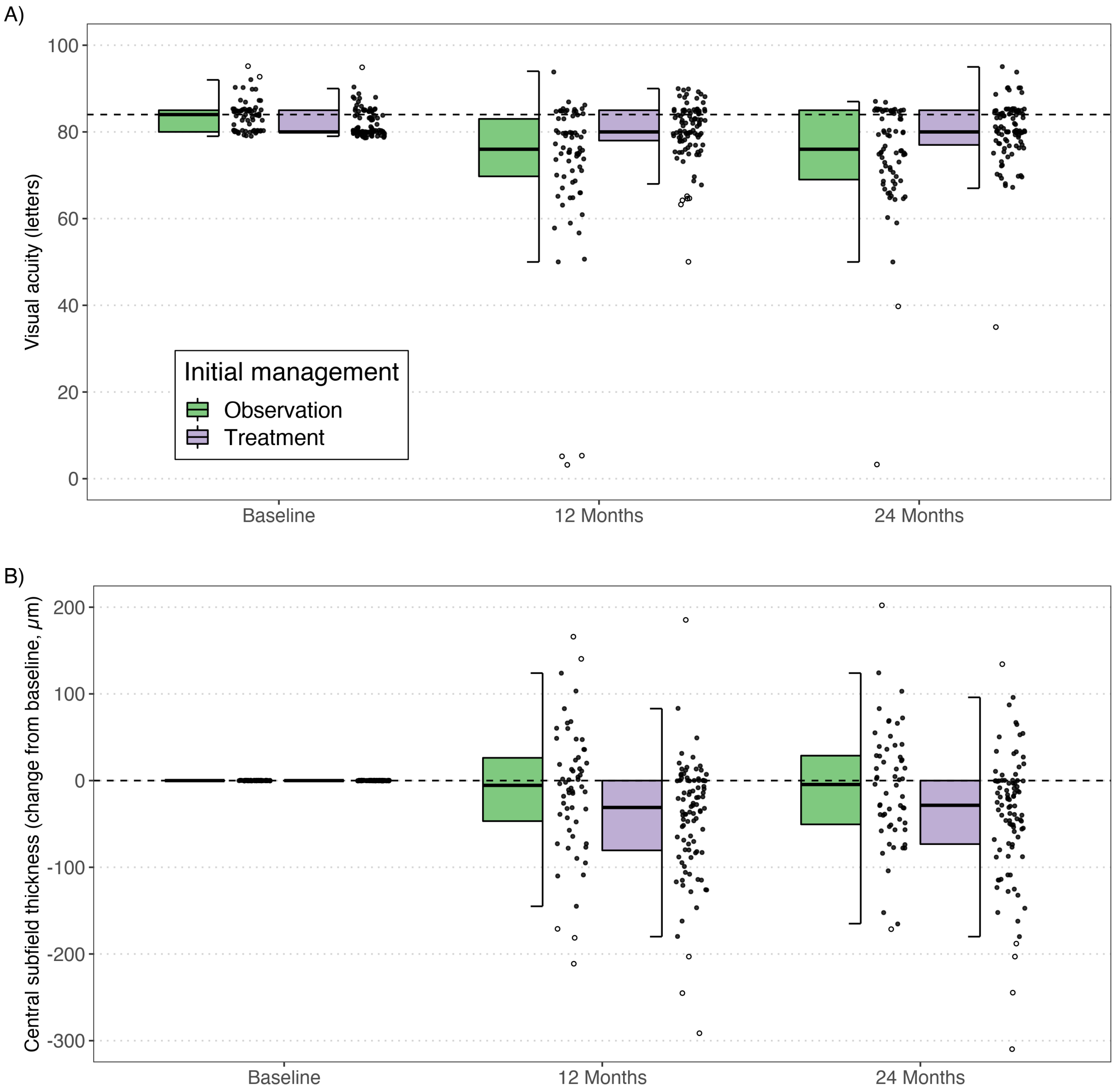

Figure S1. (A) Visual acuity and (B) change in central subfield thickness at 24 months when only eyes with center-involving diabetic macular edema are included. For each box-and-whisker plot with jitter, the horizontal bar within the box represents median; top and bottom of box, interquartile range; Upper and lower whisker extends to the closest observed data point below the upper or above the lower quartile plus 1.5 times the interquartile range. Jitters and Outlying values are plotted as black spots and circles, respectively. Values in panel A at or above the horizontal dashed line (84 letters) represent visual acuity of 20/20 or better.
