# Supplementary material for: Initial observation or treatment for diabetic macular oedema with good visual acuity: two‐year outcomes comparison in routine clinical practice: data from the Fight Retinal Blindness! Registry
Source: Acta Ophthalmol. 2020 Nov 16;100(3):285–94. doi: 10.1111/aos.14672 (PMC9290829; doi:10.1111/aos.14672)
Supplement: Supplementary file 2 — Fig. S2. Line graphs showing (A) the mean predicted visual acuity in logMAR letters; (B) the difference in the mean change in VA between initial observation (pink) and initial treatment (green); (C) the mean predicted central subfield thickness (CST, solid lines) and (D) the difference in mean change in CST between initial observed and treated eyes over 24 months when only eyes with center‐involving diabetic macular edema are included. [file AOS-100-285-s005.pdf]

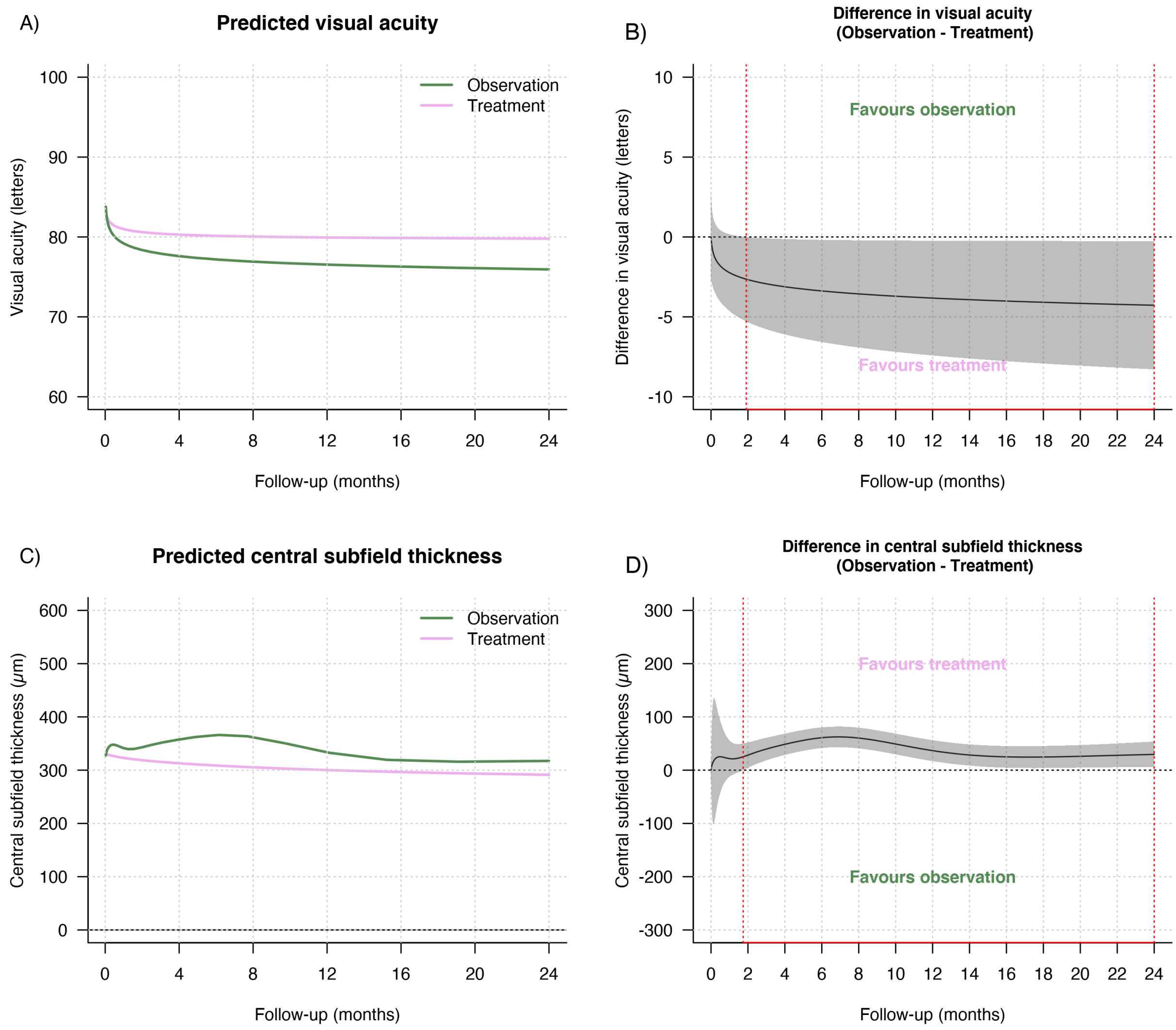

Figure S2. Line graphs showing (A) the mean predicted visual acuity in logMAR letters; (B) the difference in the mean change in VA between initial observation (pink) and initial treatment (green); (C) the mean predicted central subfield thickness (CST, solid lines) and (D) the difference in mean change in CST between initial observed and treated eyes over 24 months when only eyes with center-involving diabetic macular edema are included. The grey shaded area in figures B and D represents the 95% confidence interval. Red dashed lines in B and D indicate areas where the 95% confidence interval does not intersect with 0.
