# Supplementary material for: Initial observation or treatment for diabetic macular oedema with good visual acuity: two‐year outcomes comparison in routine clinical practice: data from the Fight Retinal Blindness! Registry
Source: Acta Ophthalmol. 2020 Nov 16;100(3):285–94. doi: 10.1111/aos.14672 (PMC9290829; doi:10.1111/aos.14672)
Supplement: Supplementary file 3 — Fig. S3. Kaplan–Meier plots for time to first treatment, intravitreal injection and laser photocoagulation in the initial observation group when only eyes with center‐involving diabetic macular edema are included. [file AOS-100-285-s004.pdf]

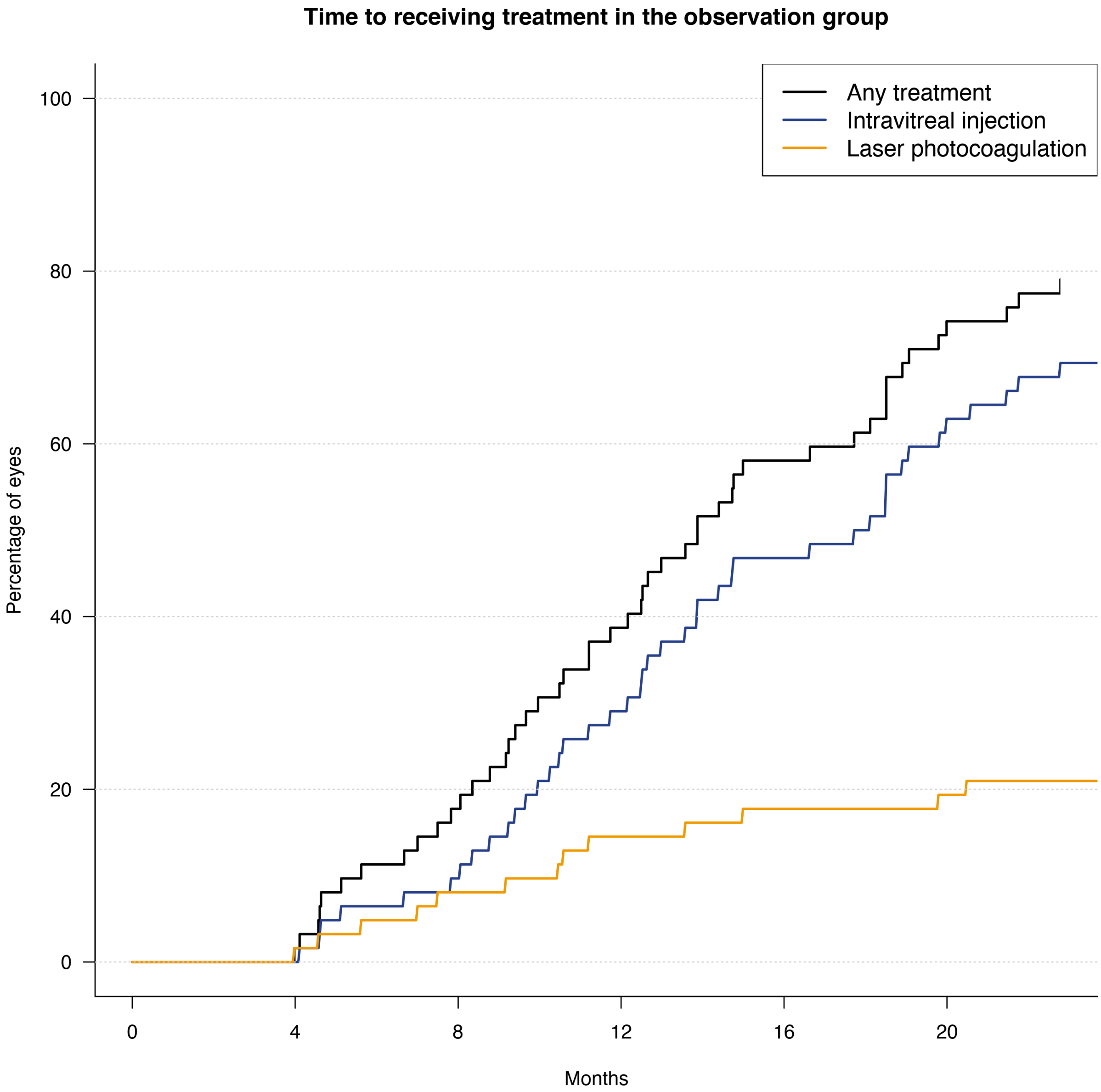

Figure S3. Kaplan-Meier plots for time to first treatment, intravitreal injection and laser photocoagulation in the initial observation group when only eyes with center-involving diabetic macular edema are included.
