# Supplementary material for: Initial observation or treatment for diabetic macular oedema with good visual acuity: two‐year outcomes comparison in routine clinical practice: data from the Fight Retinal Blindness! Registry
Source: Acta Ophthalmol. 2020 Nov 16;100(3):285–94. doi: 10.1111/aos.14672 (PMC9290829; doi:10.1111/aos.14672)
Supplement: Supplementary file 4 — Table S1. Baseline characteristics of the study groups when only eyes with center‐involving diabetic macular edema are included. [file AOS-100-285-s003.docx]

| **Table S1.** Baseline characteristics of the study groups when only eyes with center-involving diabetic macular edema are included. | | | | |
| --- | --- | --- | --- | --- |
|  | Overall | Initial observation | Initial treatment | P value |
| Eyes, n | 108 | 62 | 46 |  |
| Patients, n | 95 | 52 | 45 |  |
| Female, n (%) | 32 (34) | 21 (40) | 12 (27) | 0.13 |
| Age years, mean (SD) | 59 (12) | 61 (10) | 56 (13) | **0.03** |
| Type 1 Diabetes, n (%) | 12 (13) | 2 (4) | 10 (22) | **<0.01** |
| Diabetes duration years, mean (SD) | 14 (9) | 12 (8) | 15 (11) | 0.12 |
| Lens status (phakic), n (%) | 97 (90) | 57 (92) | 40 (87) | 0.60 |
| Diabetic Retinopathy grades, % |  |  |  | 0.20 |
| Mild NPDR | 18 | 17 | 20 |  |
| Moderate NPDR | 44 | 42 | 48 |  |
| Severe NPDR | 31 | 37 | 22 |  |
| PDR – Low Risk | 5 | 3 | 7 |  |
| PDR – High Risk | 2 | 1 | 3 |  |
| Visual acuity logMAR letters, mean (SD) | 83 (4) | 84 (4) | 82 (3) | **<0.01** |
| Central subfield thickness μm, mean (SD) | 332 (70) | 322 (67) | 342 (72) | 0.17 |
| Type of DME, % |  |  |  | - |
| Center-involving CSDME | 100 | 100 | 100 |  |
| Non center-involving CSDME | 0 | 0 | 0 |  |
| Initial management, n (%) |  |  |  |  |
| Observation | 62 (57) | 62 (100) | - |  |
| Bevacizumab | 6 (6) | - | 6 (13) |  |
| Ranibizumab | 20 (19) | - | 20 (43) |  |
| Aflibercept | 7 (6) | - | 7 (15) |  |
| Dexamethasone implant | 1 (1) | - | 1 (2) |  |
| Macular laser photocoagulation | 12 (11) | - | 12 (27) |  |
| n – Number, SD – Standard Deviation, NPDR – Non-Proliferative Diabetic Retinopathy, PDR – Proliferative Diabetic Retinopathy, VA – Visual Acuity (logMAR letters), CST – Central Subfield Thickness, DME – Diabetic Macular Edema, CSDME – Clinically Significant Diabetic Macular Edema  Significant p-values are highlighted in bold. | | | | |
