# Supplementary material for: Initial observation or treatment for diabetic macular oedema with good visual acuity: two‐year outcomes comparison in routine clinical practice: data from the Fight Retinal Blindness! Registry
Source: Acta Ophthalmol. 2020 Nov 16;100(3):285–94. doi: 10.1111/aos.14672 (PMC9290829; doi:10.1111/aos.14672)
Supplement: Supplementary file 5 — Table S2. 24‐month outcomes when only eyes with center‐involving diabetic macular edema are included. [file AOS-100-285-s001.docx]

| **Table S2.** 24-month outcomes when only eyes with center-involving diabetic macular edema are included. | | | | |
| --- | --- | --- | --- | --- |
|  | Observed data | | Observation *vs.* Treatment  Adjusted group comparison | |
| Outcomes | Initial observation | Initial treatment | Odds ratio, mean difference or ratio (95% CI) | P Value |
| Primary outcomes |  |  |  |  |
| ≥5-letter VA loss, n (%) | 43 (69) | 20 (44) | 2.4 (0.8, 6.8) ^†^ | 0.11 |
| Secondary Outcomes |  |  |  |  |
| Baseline VA letters, mean (SD) | 84 (4) | 82 (3) |  |  |
| Baseline VA Snellen equivalent, mean | 20/25 | 20/25 |  |  |
| VA change from baseline to 24 months letters, crude mean (95% CI) | -10 (-13, -7) | -4 (-7, 0) |  | **<0.01** |
| VA change from baseline to 24 months letters, adjusted mean (95% CI) | -7 (-10, -40 | -3 (-6, 0) | -4 (-8, 0) ^‡^ | 0.065 |
| ≥10-letter loss from baseline, n (%) | 25 (40) | 10 (22) | 5.3 (1.3, 21.0) ^†^ | **0.018** |
| ≥15-letter loss from baseline, n (%) | 18 (29) | 2 (4) | 18.2 (3.0, 113) ^†^ | **<0.01** |
| VA at 24 months letters, mean (SD) | 74 (13) | 79 (9) |  |  |
| VA at 24 months Snellen equivalent, mean | 20/32 | 20/32 |  |  |
| ≥ 84 letters (20/20 or better), n (%) | 17 (27) | 14 (30) | 0.8 (0.2, 2.3) ^†^ | 0.62 |
| ≥ 69 letters (20/40 or better), n (%) | 47 (76) | 42 (91) | 0.1 (0.0, 0.8) ^†^ | **0.026** |
| Baseline CST μm, mean (SD) | 322 (67) | 342 (72) |  |  |
| CST change from baseline to 24 months μm, crude mean (95% CI) | -13 (-33, 8) | -37 (-59, -15) |  | 0.11 |
| CST change from baseline to 24 months μm, adjusted mean (95% CI) | -20 (-40, 0) | -46 (-65, -27) | +26 (2, 50) ^‡^ | **<0.01** |
| Injections, median (Q1, Q3) | 7 (3, 10) | 6 (3, 12) | 1.1 (0.8, 1.4) ^§^ | 0.76 |
| Proportion of eyes receiving any treatment in the observation group | 49 (79) |  |  |  |
| Proportion of eyes receiving at least one injection in the observation group, n (%) | 43 (69) |  |  |  |
| Time until first injection days, median (Q1, Q3) | 545 (322, 903) |  |  |  |
| VA at first injection letters, mean (SD) | 72 (11) |  |  |  |
| VA change at first injection letters, mean (SD) | -12 (11) |  |  |  |
| < 5-letters loss, n (%) | 14 (23) |  |  |  |
| ≥ 5 and < 10-letters loss, n (%) | 7 (11) |  |  |  |
| ≥ 10 letters loss, n (%) | 22 (36) |  |  |  |
| Proportion of eyes receiving at least one laser photocoagulation in the observation group, n (%) | 13 (21) |  |  |  |
| Time until first laser days, median (Q1, Q3) | 602 (213, 1078) |  |  |  |
| VA at first laser letters, mean (SD) | -8 (10) |  |  |  |
| Laser photocoagulation sessions, median (Q1, Q3) | 0 (0, 0) | 0 (0, 1) | 0.6 (0, 24) ^§^ | 0.79 |
| Visits, median (Q1, Q3) | 17 (12, 22) | 12 (8, 18) | 1.6 (1.3, 2.0) ^§^ | **<0.01** |
| CI – Confidence Interval, VA – Visual Acuity, n – Number, SD – Standard Deviation, CST – Central subfield thickness, Q1 – First Quantile, Q3 – Third Quantile.  ^†^ calculated from logistic mixed-effects regression models adjusting for age, VA/CST, lens status, grade of diabetic retinopathy at baseline (fixed effects), and practice and intra-patient correlation for bilateral cases (random effects).  ^‡^ calculated from non-linear mixed-effects regression models adjusting for age, VA/CST, lens status, grade of diabetic retinopathy at baseline (fixed effects), and practice and intra-patient correlation for bilateral cases (random effects).  ^§^ adjusted ratio (95% CI) of number of injections, laser photocoagulations or visits between observation *vs.* treatment group. It was calculated from Poisson regression models adjusting for age, VA/CST, lens status, grade of diabetic retinopathy at baseline (fixed effects), and practice and intra-patient correlation for bilateral cases (random effects).  Significant p-values are highlighted in bold. | | | | |
